# A New Set of SSR Markers Combined in One Reaction for Efficient Genotyping of the Hexaploid European Plum (*Prunus domestica* L.)

**DOI:** 10.3390/plants14152281

**Published:** 2025-07-24

**Authors:** Jana Čmejlová, Kamila Pluhařová, Boris Krška, Radek Čmejla

**Affiliations:** Research and Breeding Institute of Pomology Holovousy Ltd., Holovousy 129, 508 01 Holovousy, Czech Republic; jana.cmejlova@vsuo.cz (J.Č.); boris.krska@vsuo.cz (B.K.)

**Keywords:** European plum, *Prunus domestica* L., SSR marker, genotyping, fragment analysis

## Abstract

The European plum (*Prunus domestica* L.) is a hexaploid species that is grown worldwide for its tasty fruits. Many pomological forms and varieties exist, and thus it is important for genebank curators, breeders, growers, and/or control authorities to distinguish them with certainty. The purpose of this study was to select and verify a set of simple sequence repeat (SSR) markers for reliable genotyping, and to optimize their use in a one-reaction format for easy routine practice. After testing 78 SSR markers from different diploid *Prunus* species, 8 SSR markers were selected, multiplexed, and successfully verified as being able to distinguish all 242 unique genotypes tested. The selected markers were relatively easily scored and highly heterogenic, giving more than 35 alleles/genotype on average. The allele atlas was created to become a valuable tool for allele calling that should lead to standardized and reliable genotyping results between laboratories. The population analysis confirmed high diversity of the Czech germplasm collection used. The kit was also successfully tested for diploid “plums” of various origins and interspecies hybrids, as these are sometimes phenotypically indistinguishable from hexaploid European plums. The one-tube approach substantially simplified the plum genotyping laboratory workflow, minimizes errors, and saves labor, time, and money.

## 1. Introduction

The European plum (*Prunus domestica* L.) is an agronomically important hexaploid species (2n = 6× = 48) from the Rosaceae family, cultivated in temperate areas worldwide for its popular fruits. Beyond European plums, closely related and phenotypically similar diploid species (2n = 2× = 16) also exist, and they are important in both commercial cultivation and breeding programs due to their diverse characteristics and adaptability. They comprise, for example, *Prunus salicina* Lindl. (Chinese plum or Japanese plum) with large, juicy fruits commonly used for fresh consumption; *Prunus cerasifera* Ehrh. (cherry plum or myrobalan) that is often used as a rootstock for other plum varieties; or *Prunus americana* Marsh. (American plum) that may be a part of breeding programs for its hardiness. The whole genus *Prunus* contains many highly genetically related species and a lot of them bear commercially important fruits, for example, sweet and sour cherries (*P. avium* L. and *P. cerasus* L., respectively), apricots (*P. armeniaca* L.), peaches (*P. persica* L.), almonds (*P. dulcis* Batsch), Japanese plums (*P. salicina* Lindl.), or European plums (*P. domestica* L.), among others. These species have different ploidy (2n = 2× = 16 in sweet cherry, apricot, peach, and Japanese plum; 2n = 4× = 32 in sour cherry; 2n = 6× = 48 in European plum) [1]. Moreover, many interspecific hybrids also exist [2]. In particular, *Prunus salicina* L. is often crossed with other diploid *Prunus* species, especially apricot (*Prunus armeniaca* L.) [3]. Furthermore, interspecific hybrids between hexaploid European plum and diploid apricot have also been reported [4].

All these commercially important fruit crops have been bred into many varieties with unique characteristics. Being vegetatively propagated, it is possible to identify each variety based on its DNA profile by genotyping (colloquially DNA fingerprinting). Genotyping of fruit crops is usually exploited for scientific purposes (e.g., characterization of germplasm collections or evaluation of genetic diversity). Nonetheless, it may be also used in the commercial sphere for the verification of varieties during, e.g., registration renewals, verification of the identity of propagated and sold plant material, controlling license agreement compliance, etc. However, the price per analysis is an important factor in the availability of this method.

Modern genotyping techniques more and more often utilize single nucleotide polymorphisms and high-throughput sequencing. This approach is not appropriate for the routine genotyping of European plums; however, due to their hexaploid genome [5], since high-sequence coverage is necessary in order to not miss some alleles, requiring the use of advanced biostatistics leads to a high cost per analysis. As an alternative, co-dominantly inherited simple sequence repeat (SSR) markers, also known as microsatellites, offer a cheaper and reliable genotyping approach and are in fact the number one choice for fingerprinting not only in plants [6]. SSRs are defined by the presence of several sequence repeat units containing up to 8 bp; however, microsatellites with di-nucleotide repetition are the most frequently used molecular markers for plant genotyping fruit crops including [6]. For reliable scoring and consistent reproducibility, a good SSR marker should be highly polymorphic, specifically PCR-amplified, and preferentially exhibit a low level of stuttering that is caused by polymerase slipping during PCR amplification [7]. Depending on the informative content of a marker and the genetic diversity of the crop, usually twelve or fewer microsatellites have been found to be sufficient for distinguishing varieties resulting from sexual reproduction, including self-pollination [6]. The length of an SSR locus representing a particular allele is usually determined by the fragment analysis of the respective PCR amplicon using capillary electrophoresis with the ability to distinguish between alleles differing by only one nucleotide. Another advantage is the possibility to analyze several markers in one multiplex PCR/fragment analysis reaction to lower the cost per analysis of one genotype. Multiplexing may be achieved by the use of differently fluorescently labeled marker-specific primers, and/or by a combination of different fragment lengths in one reaction.

Several studies examining hexaploid European plum genotyping by SSR markers have been published [8,9,10,11,12,13,14,15]. In 2020, the European Cooperative Programme for Plant Genetic Resources (ECPGR) recommended a set of nine SSR markers amplified in separate PCR reactions for European plum genotyping [16], and the protocol has subsequently been used for plum fingerprinting in Lithuania [17] and Norway [18]. All these studies adopted different SSR markers described in the diploid *Prunus* species, such as peach (for example, the UDP set [19], the CPPCT set [20], the BPPCT set [21]); Japanese plums (the CPSCT set [22]); sweet cherry (the EMPA set [23]); and almond (the CPDCT set [24]), because the development and validation of native SSR markers in hexaploid European plum are difficult. A complication may be that primers designed for other *Prunus* species are not always usable in *P. domestica* L. [14]; therefore, it is necessary to test not only the efficiency of PCR amplification of the target locus, but also the ease of evaluation of fragment analysis outputs. Some markers show such high stuttering that it hampers reliable evaluations of marker allele compositions, especially in hexaploid organisms, in which alleles may be present in numerous copies per genome, further complicating the interpretation of results and lab-to-lab transferability.

The aim of this study was to provide easily scorable and highly heterogenic SSRs with low stuttering, enabling reliable cross-laboratory plum genotyping. A further ambition was the development of a kit that would combine the selected microsatellites into one reaction to simplify the whole process and to lower the cost per analysis. A consistent evaluation of fragment analysis outputs and clonal variant identification were additional aims of the work. In parallel, diversity of the Czech plum germplasm collection was analyzed. As hexaploid and diploid plum-like species and their interspecific hybrids are sometimes phenotypically indistinguishable, the kit was also tested in these samples.

## 2. Materials and Methods

### 2.1. Plant Material, DNA Isolation

Unique accessions (n = 242) from a plum germplasm collection maintained in the Research and Breeding Institute of Pomology Holovousy (Location: 50°22′46′′ N, 15°34′33′′ E), which contains also main European and North American varieties, were used in the study (Supplementary Table S1). DNA was isolated from 100 mg of young leaves homogenized in liquid nitrogen by an ExgeneTM Plant SV mini kit (GeneAll Biotechnology, Seoul, Republic of Korea) according to the manufacturer’s instructions. DNA concentration and quality were assessed by a NanoDrop Lite Spectrophotometer (Thermo Fisher Scientific, Waltham, MA, USA), and DNA was diluted to 10 ng/µL for further analysis.

### 2.2. Choice of SSR Markers and Their Evaluation, Fragment Analysis, and Sequencing

Seventy-eight SSR markers originating from different *Prunus* species, either previously used in European plum or highly heterogenic in other *Prunus* species, were chosen for primary testing. PCR primers for their amplification were adopted from original publications ([19,20,21,22,23,24,25,26,27,28,29,30,31,32,33,34,35]; Supplementary Table S2). A small set of eight unrelated *Prunus domestica* L. varieties (‘Topfirst’, ‘Wangenheimova’, ‘Nancyská’, ‘Hanita’, ‘Lováňská’, ‘Stanley’, ‘Švestka domácí’, and ‘Valor’; their characteristics are listed in Supplementary Table S1) was used to first test the PCR amplification efficiency of each marker using the following PCR protocol: 5 μL of Phusion Flash High-Fidelity PCR Master Mix (Thermo Fisher Scientific), 1 μL of each primer (final concentration 0.2 μM, one of them fluorescently labeled), 2 μL DNA (10 ng/μL), and PCR grade water up to 10 μL. PCR was run on a C1000 PCR cycler (Bio-Rad, Hercules, CA, USA) using a universal temperature profile as follows: 98 °C/1 min; 23 cycles (98 °C/10 s, 58 °C/10 s, 72 °C/15 s); final extension 72 °C/15 s. Afterward, 1 μL of the PCR product was mixed with 15 μL Hi-Di Formamide and 0.5 μL GeneScan 600 LIZ dye Size Standard v2.0 (both Thermo Fisher Scientific). Samples were denatured at 95 °C for 2 min and run on a 3500 Genetic Analyzer (Thermo Fisher Scientific). Results were analyzed in v5 GeneMapper software (Thermo Fisher Scientific). Two to three PCR cycles were added if a weak signal or no signal was observed.

The following criteria were evaluated to exclude a candidate SSR marker from further analyses: (i) no or very inefficient amplification; (ii) low heterogeneity (less than 3.5 different alleles/genotype on average); (iii) more than six fragments observed in any hexaploid genotype tested; (iv) high stuttering of alleles, complicating proper allele-scoring; (v) observation of alleles differing in less than 1.0 nucleotide, making bin definition impossible using GeneMapper software; and (vi) ease of allele-scoring, assessed by two experienced persons and classified from 1 (easy) to 5 (very complicated).

SSR markers that passed the criteria were chosen for a second round of analysis in another 41 genotypes of *Prunus domestica* L. (Supplementary Table S1). At this step, heterogeneity characteristics of selected SSR markers were also evaluated. A so-called “allelic phenotype” approach [14], which means scoring and recording an allele only as present/absent, was used. To distinguish between the real allele frequency and observed allele “frequency” in organisms with ploidy higher than two, the term allele “occurrence” instead of “frequency” is used herein for hexaploid plums. The following parameters were calculated for each marker: (i) the number of alleles; (ii) the number of rare alleles (with occurrence below 5%); (iii) the average number of alleles per genotype; (iv) the size of the shortest and longest alleles (nucleotides); and (v) the presence of alleles differing by 1 nucleotide to allow successful binning. Based on these criteria, the best candidate markers were selected for a final evaluation with the aim of combining as many markers as possible in one reaction.

For this purpose, the respective primers were labeled by 6-FAM, VIC, NED, or PET, and concentrations of primers in a multiplex fragment analysis were fine-tuned to obtain comparable signal heights for all markers. The multiplex PCR reactions were run under the same conditions as the simplex ones but with 25 PCR cycles. If alleles with a low signal were identified, indicating a possible mutation in a primer-annealing region, primers surrounding the amplified fragment were designed based on the *Prunus domestica* Draft Genome Assembly v1.0 & Annotation v1.0.a1 [36] to check primer complementary sequences. PCR-generated fragments were agarose gel separated, cut out, purified by a GeneAll Expin Combo GP kit (GeneAll Biotechnology), and sequenced by a BigDye™ Terminator v3.1 Cycle Sequencing Kit (Thermo Fisher Scientific) according to the manufacturers’ instructions. Identified mutations were taken into account for the final design of the affected primers; Geneious Prime^®^ software version 2024.0 (GraphPad Software LLC d.b.a Geneious, Boston, MA, USA) was used for sequence analysis. Finally, all alleles were verified as belonging to a particular marker by a simplex PCR and fragment analysis. The final kit was tested on the remaining germplasm accessions to obtain detailed statistics for each marker (number of alleles, allele occurrence, number of alleles occurring in <5%, average/total number of alleles per genotype, allele combinations and unique allele combinations, allele range, and allele distribution in markers). All samples were tested minimally twice. A discrimination potential was used as an indirect parameter to show how many unique genetic profiles could be, in theory, obtained if markers were used in combinations. This was calculated as the product of the number of observed allele combinations for each marker.

### 2.3. Genetic Structure Analysis

The genetic structure of the analyzed population was determined by the Bayesian model-based clustering method in Structure v. 2.2.4 software [37]. Absent alleles in individuals with less than six allelic variants per locus were uploaded as missing data (marked −9). Ten independent runs were conducted for each K (1–26) for all samples from Supplementary Table S1 with a burn-in length of 200,000 and 500,000 MCMC reps after burn-in. Structure Harvester version 0.6.1 [38], which implements the method developed by [39], was used to evaluate K values for the analyzed data. Varieties with inferred clusters ≥0.8 and ≥0.9 for two groups with the highest delta K values, respectively, were assumed as reference varieties for individual groups.

POLYGENE software version V1.7 [40] was used to analyze relationships in a population of 242 unique genotypes. Hierarchical clustering was performed using the unweighted pair group method with arithmetic mean (UPGMA) [41].

### 2.4. Kit Validation

To validate the kit, 54 blind leaf samples from a commercial plum grower were analyzed and compared with germplasm accessions for their identity. DNA isolation and analyses were performed as described above. Afterwards, results were compared with the information provided by the grower. A parentage analysis was performed for selected varieties to verify their known parents using the POLYGENE software.

## 3. Results

### 3.1. Selection of Reliable SSR Markers

Seventy-eight previously published SSR markers, including nine recommended by the ECPGR, were selected (Supplementary Table S2). All markers had a 2 nt repetition unit. The first screening was focused on the ability to efficiently PCR amplify each marker in eight hexaploid *P. domestica* L. genotypes. Supplementary Table S2 summarizes the results for each marker and shows typical examples of fragment analysis outputs. In this step, 11 SSR markers failed to provide a clear PCR amplicon, even after three additional PCR cycles (Supplementary Table S2; n = 11; e.g., AMPA105). In the remaining SSRs, markers presenting more than six alleles in at least one genotype were excluded (n = 7; for example, BPPCT008). The exclusion of markers with low heterogeneity, i.e., less than 3.5 different alleles per genotype on average, followed (n = 13; e.g., EPPISF001). In markers with higher heterogeneity (n = 47), closely migrating alleles were searched for, and true allele calling (i.e., binning efficiency) was evaluated. Markers with co-migrating peaks were discarded (marked as “binning impossible”; n = 3 in 47 remaining SSRs, e.g., UDP98-410), as were those with alleles differing in less than one nucleotide (marked as “binning problematic”; n = 14, i.e., BPPCT004). In the remaining 28 SSR markers, the stuttering of alleles and ease of their evaluation were finally taken into account. Some stuttering was observed in nearly all SSR markers, the exceptions were EMPAS02 and UCD-CH17. Longer alleles usually showed a higher stutter and lower signal in comparison with shorter ones. Finally, the ease of scoring was evaluated by two experienced persons, and 13 SSR markers with ease scores of “mark 1” were selected for the second round of analyses.

Statistical parameters characterizing the genotyping potential of these markers were evaluated using another 41 genotypes of *Prunus domestica* L. (Supplementary Tables S1 and S2). An average number of different alleles per genotype was used as a main criterion for evaluating marker heterogeneity. Combining all of these criteria, the UCD-CH13 marker was excluded due to lower heterogeneity (averagely 3.96 alleles per genotype) in comparison with other markers, exhibiting 4.24–5.29 alleles per genotype on average (Supplementary Table S2). The remaining 12 microsatellites were finally selected for next analyses (Table 1), and the set also included two ECPGR-recommended markers. The remaining ECPGR markers were excluded for various reasons (Table 2).

### 3.2. Multiplexing of the Selected SSR Markers

The next step was focused on combining as many selected SSR markers as possible in one multiplex reaction, ideally sufficient to reliably differentiate all germplasm accessions. Based on the lengths of amplified fragments as the main multiplexing criterion, eight markers were suitable for combinations into the final multiplex. However, the primers for CPPCT033 needed to be redesigned to shorten the amplified fragments and concurrently to increase the annealing temperature of this primer pair to work well under multiplex PCR conditions. Since the positions of the primers changed, the marker was renamed “CPPCT033_a” to distinguish it from the original one; however, the same region of a plum genome was amplified. After that, it was possible to combine two markers in one detection channel with a sufficient gap between them for unequivocal marker allele calling; this multiplex was called 8-plex (Table 1). The remaining four markers (CPSCT026, EMPAS14, UCD-CH17, and UDAp446) were also successfully tested in a multiplex configuration (4-plex) without amplification artefacts.

**Figure 1:**
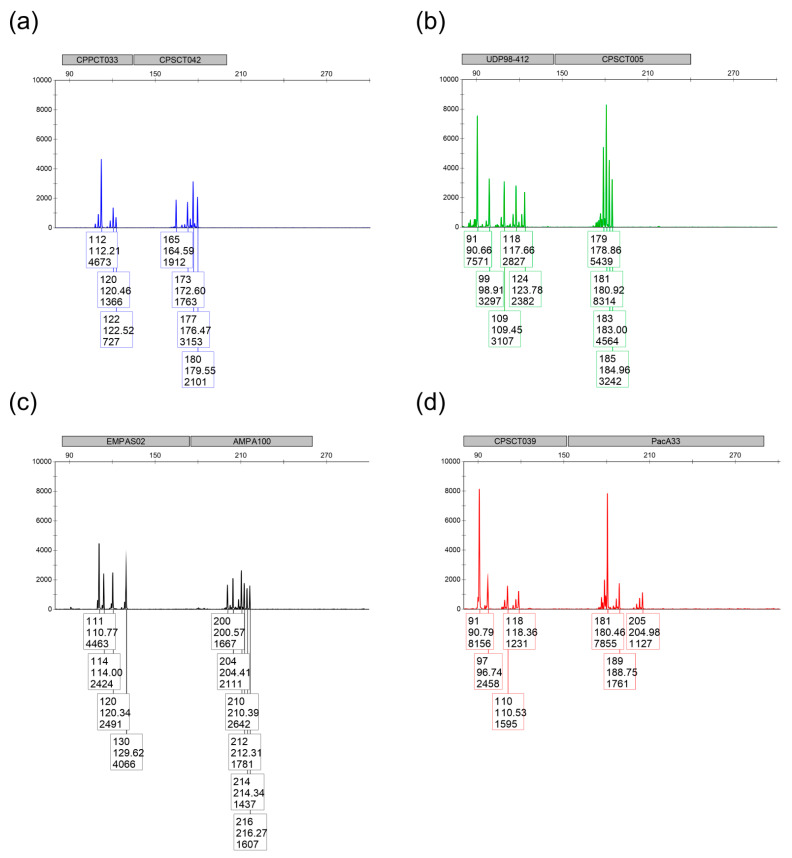
Representative output for 8-plex in the ‘Albatros’ variety. (**a**) Blue detection channel (6-FAM label): CPPCT033_a + CPSCT042; (**b**) Green detection channel (VIC label): UDP98-412 + CPSCT005; (**c**) Yellow detection channel (NED label): EMPaS02 + AMPA100; (**d**) Red channel (PET label): CPSCT039 + PacA33.

**Figure 2:**
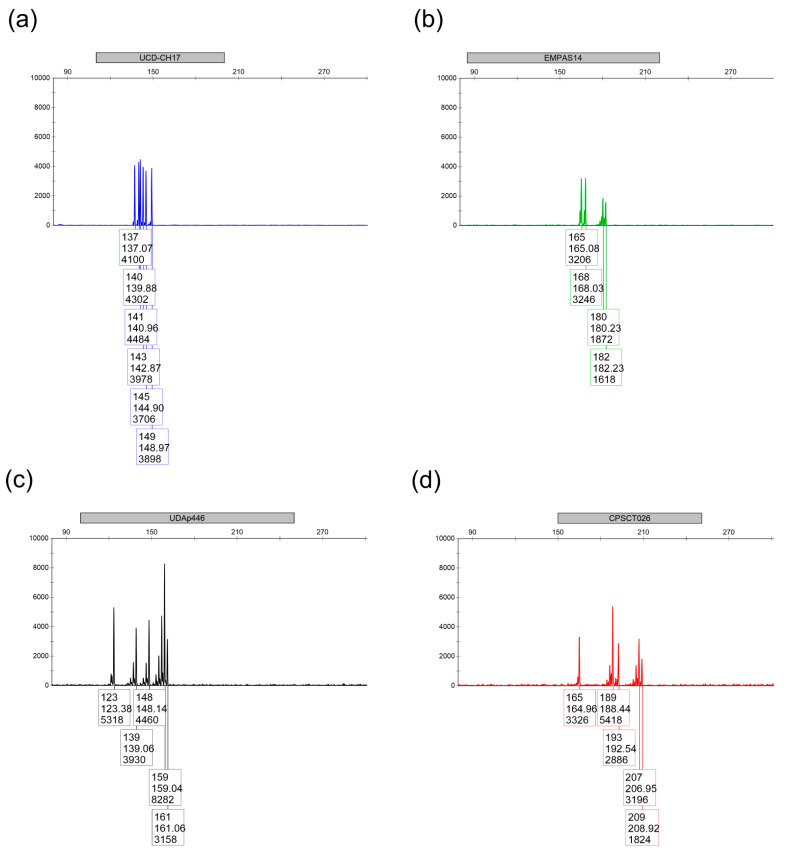
Representative output for 4-plex in the ‘Albatros’ variety. (**a**) Blue detection channel (6-FAM label): UCD-CH17; (**b**) Green detection channel (VIC label): EMPAS14; (**c**): Yellow detection channel (NED label): UDAp446; (**d**) Red channel (PET label): CPSCT026.

If a low signal was obtained for any allele, the target sequences of the PCR primers were verified by sequencing. Thus, the sequence of several primers had to be modified, or an extra primer added (in the case of UDP98-412), to better detect all plum marker alleles. Finally, the concentration of each primer was optimized to obtain approximately the same signal height for alleles in all markers (Table 3). However, in spite of these modifications, signals remained low for some alleles (Supplementary Table S3). Examples of fragment analysis outputs for 8-plex and 4-plex are shown in Figure 1 and Figure 2, respectively.

### 3.3. Stuttering of SSR Markers

The allele’s stuttering intensity was one of the many parameters analyzed for suitable marker selection, as SSR markers with all alleles showing only one distinct peak (i.e., no stutter) seem to be rare. Only two markers (EMPAS02, UCD-CH17) presented in all alleles without stutter peaks. Other selected markers exhibited some allele stuttering, but at manageable levels. Such a stutter usually appeared in longer alleles and increased with allele lengths; however, in the CPSCT042 marker, the opposite was observed—the shortest allele 143 had the highest stutter. To promote correct allele calling in selected markers, the typical stutter pattern of individual alleles and their combinations with neighboring alleles were determined and served as a useful tool during the scoring of difficult genotypes (Allele atlas; Supplementary Table S3).

### 3.4. Statistics of Selected SSR Markers in Hexaploid European Plums

In total, 242 unique hexaploid accessions were analyzed by the 12 selected SSR markers (Supplementary Table S1). Based on the results, statistics for each marker were calculated (Table 4). The average number of alleles per marker/genotype ranged from 4 to 5.2. In the CPPCT033 marker, only one allele was recorded in one sample; otherwise, at least two alleles were observed in all other markers in all samples (Supplementary Table S4).

The highest discrimination potential was found for the PacA33 marker: 40 alleles produced 217 combinations, of which 197 were unique, implying this marker alone was able to discriminate 81.4% of all samples. The combination of eight selected multiplexed markers (8-plex; CPPCT033_a, CPSCT042, UDP98-412, CPSCT005, EMPaS02, AMPA100, CPSCT039, and PacA33) was sufficient to discriminate between all genotypes, and 4-plex markers were redundant. In fact, five markers were able to tell apart 97% of all genotypes, and the combination of all eight markers was only needed for the eight remaining samples (Table 5). Based on the number of observed allele combinations in these eight markers (Table 4), up to 5.06 × 10^17^ unique genetic profiles may be, in theory, possible (discrimination potential). If all 12 markers were taken into account, only UDAp446 from the 4-plex was helpful (though not necessary) for the identification of three genotypes. When combining all 12 markers, however, the discrimination potential increased to a theoretical 4.78 × 10^26^ unique genetic profiles.

**Figure 3:**
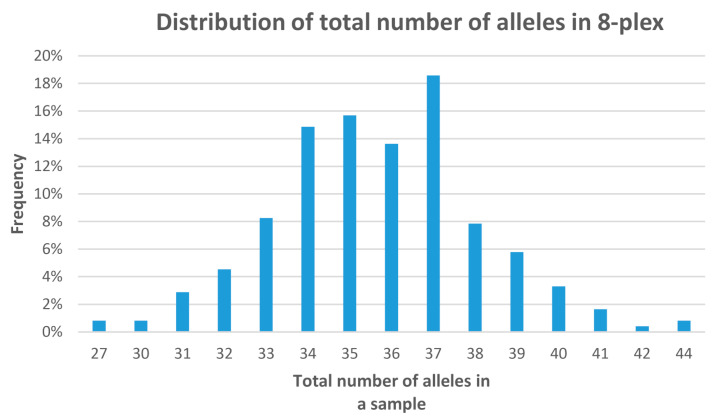
Distribution of the total number of alleles in samples for 8-plex.

Overall, 8-plex showed 35.7 alleles/genotype on average (with median 36.0; range 27 to 44 out of 48 possible maximum; the mode was 37 alleles found in nearly 19% of samples; Figure 3); 8-plex + 4-plex exhibited 54.5 alleles/genotype on average (with median 55.0; range 43 to 64 out of 72 possible maximum; 54 or 55 alleles were observed most frequently, both 11.8%). Detailed allele distributions in markers, their comparison, and allele occurrences for each marker may be found in Supplementary Table S4.

### 3.5. Genetic Structure of Hexaploid Plum Samples

An analysis of the genetic structure of the hexaploid plum population resulted in a distribution of samples into 20 and 6 groups with the highest delta K of 85,3 and 37,2, respectively (labeled K20.1 to K20.20, and K6.1 to K6.6; Supplementary Table S5). The reference varieties for K6.1 included greengages known for centuries [‘Wazonova’, ‘Oullinská renklóda’ (‘Reine Claude d’Oullins’), and ‘Zelená renklóda’ (‘Reine Claude Verte’)]; K6.2 may be represented by the long-grown variety ‘Viktoria’; and varieties of the K6.3 groups were derived from homeside plums. K6.4 represented local varieties, and K6.5 varieties were related to ‘President’ (a chance seedling from the 19th century), ‘Stanley’ (‘Agen’ × ‘Grand Duke’), ‘Bluefre’ (‘President’ × ‘Stanley’), and ‘Rheingold’ (‘President’ × ‘Severn Cross’). K6.6 exhibited only one reference variety: ‘Ruth Gerstetter’. While the K6.6 group contained the fewest varieties (n = 21), the most represented group was K6.5 with 58 varieties. These six groups were further divided into twenty ones, e.g., the original group K6.1 of greengages (n= 46) was divided into two main groups, K20.10 (n = 13) and K20.17 (n = 23), represented by ‘Oullinská renklóda’ and ‘Zelená renklóda’, respectively. In variety ‘Wazonova’, 58.2% of its genome corresponded to K20.17 and 31.5% resembled K20.10, respectively. Similarly, the most numerous group K6.5 (n = 58) split into two main discrete sub-groups: K20.7 (n = 16) with the representative variety ‘President’, and K20.20 (n = 35) represented by ‘Stanley’. Approximately half of the genome of the varieties ‘Bluefre’ and ‘Rheingold’ corresponded to K20.7 and half to the K20.20. Representative varieties for other groups may be found in Supplementary Table S5.

During 8-plex testing, in some samples with the same name, differences were repeatedly noted in usually one marker, indicating they were likely clonal variants of the same variety (Supplementary Table S1, varieties in green). The most differences between clones were observed in the PacA33 marker (eight varieties), followed by CPSCT039 (four varieties), CPSCT005 (three varieties), and AMPA100 (two varieties); in the remaining four markers (CPPCT033_a, CPSCT042, UDP98-412, and EMPAS02), no changes were observed. Differences were of various kinds: the presence/absence of an allele; differences in length by two nucleotides; or differences in length by four and more nucleotides.

**Figure 4:**
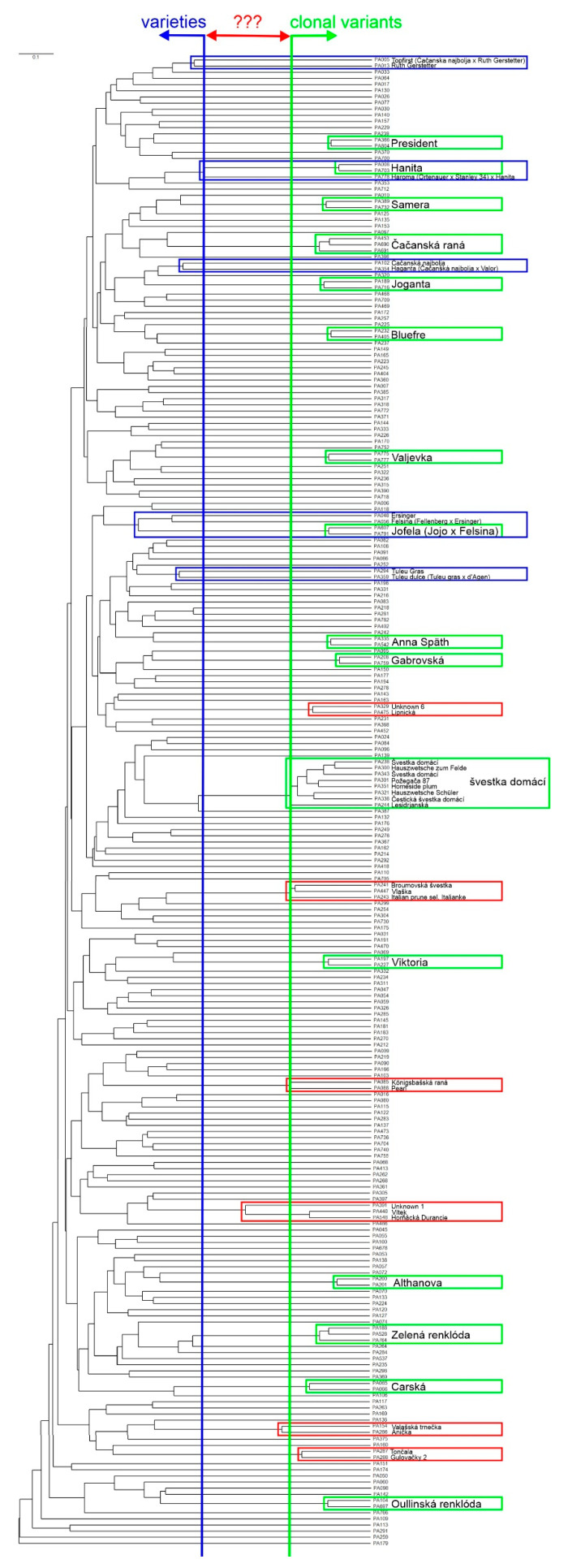
Relationships in a population of 242 unique genotypes. Green frame: clonal variants of the same variety; Blue frame: directly related different varieties; Red frame: potential clonal variants; ???: uncertain.

The relationships of the 242 unique genotypes tested were then analyzed with the aim of discriminating the actual variety from potential clonal variants. A dendrogram was then constructed using all 12 SSR markers, and two borders of the relationships were demarcated based on parent–kin knowledge and/or the same name of the variety (Figure 4). The left blue line defined the identified first-line relation branching border in the tree [e.g., ‘Ruth Gerstetter’ (PA013) is a parent of ‘Topfirst’ (PA005) and others] and varieties were situated to the left of this cut-off; the right green line delineated clonal variants of the same variety [e.g., several clonal variants of the ‘Švestka domácí’ variety, including international synonyms]. All clonal variants of the same variety tested bifurcated to the right of the green line, which also allowed the identification of possible clonal variants among old local varieties of unknown origin with their branching point near this border [e.g., ‘Valašská Trnečka’ (PA154) and ‘Anička’ (PA266)]. In general, parent–progeny and clonal variants differed in minimally 13 and maximally 2 alleles, respectively (Supplementary Table S5). It was not possible to evaluate samples on branches bifurcating in the middle of the dendrogram, as these were usually local variants of unknown origin. With maximally five different alleles, these were considered to be possible clonal variants.

### 3.6. Verification of the Kit

Fifty-four blind samples of unknown, commercially grown varieties were tested using the final 8-plex kit, and the results were compared with the genetic profiles of analyzed germplasm accessions for their identification. The genetic profiles of all samples were present in the database, and represented 11 varieties: ‘Imperial’ (4 samples); ‘Aprimira’ (syn. ‘Miracose’) (4 samples); ‘Toptaste’ (10 samples); ‘Jojo’ (15 samples); ‘Hanka’ (1 sample); ‘Joganta’ (5 samples); ‘Stanley’ (3 samples); ‘Topfirst’ (7 samples); ‘Althanova’ (2 samples); ‘Zelená renklóda’ (2 samples); and ‘Nancyská’ (1 sample). The grower confirmed that the varieties had been determined correctly.

A kinship analysis indicated a potential first-line relationship for some varieties (Figure 4, blue frames), e.g., ‘Topfirst’ (‘Čačanska Najbolja’ × ‘Ruth Gerstetter’), ‘Haganta’ (‘Čačanska Najbolja’ × ‘Valor’), and ‘Jofela’ (‘Felsina’ × ‘Jojo’). For all varieties, parents were successfully verified (Supplementary Table S5). For ‘Jofela’, two clonal variants were identified (PA607, PA791), differing in one allele in the PacA33 marker (221 vs. 207). A parentage analysis showed that allele 221 was first inherited from ‘Felsina’, and allele 207 had to occur later on due to somatic mutation. A simulation in the POLYGENE software version V1.7 confirmed these results with a zero estimated genotyping error rate (Supplementary Table S5).

## 4. Discussion

Modern genotyping methods rely on the presence of SNPs that may be detected by either SNP chips or sequencing (genotyping by sequencing). A standardized subset of several hundred SNPs (called multiple nucleotide polymorphisms, MNPs) may be used to simplify the genotyping process [42]. While these one-reaction methodologies are well suited for diploid organisms, there are substantial difficulties for polyploids, including European plums. There is no reference genome, making it hard to develop and validate an SNP set, and the presence of six chromosomal sets poses a challenge for data interpretation, risking an allele omission [43]. Moreover, if a set of SNPs was finally developed for *Prunus domestica* L. it would likely not work for other diploid “plum” species (e.g., *P. cerasifera* Ehrh., *P. salicina* L., or *P. americana* Marsh.), or their interspecific hybrids. From this point of view, the SSR markers presented in this work offer a superior alternative for European and Asian plum genotyping, applicable in routine molecular biology laboratories.

Though a couple of publications have described the use of SSR genotyping in plums [8,9,10,11,12,13,14,15], all have used different sets of SSR markers adopted for their own purposes, and none have systematically focused on providing a validated set of easily scored markers that could become a laboratory standard for routine plum genotyping. It was not until 2020 that nine SSR ECPGR-recommended markers were proposed as a standard set for genotyping European plum accessions [16]; however, these were amplified in nine separate PCR reactions.

From the practical point of view, it is very advantageous to multiplex several SSR markers into one reaction to speed up and cheapen the process; however, genotyping in one reaction is still not a standard in plants, though several examples have already been published for fruit crops, e.g., apples [44], pears [45], hazelnuts [46], strawberries [47], or blueberries [48]. Suitable markers for plum genotyping were selected from 78 SSR markers developed in different diploid species of the genus *Prunus*, including the ECPGR-recommended SSR markers. With the view of future multiplexing, all SSR markers were tested under uniform PCR conditions. The Phusion Flash High-Fidelity PCR Master Mix was chosen for amplification as this master mix contains an accurate proofreading DNA polymerase, which has a unique processivity-enhancing domain, making this polymerase accurate, rapid, and robust (Thermo Fisher Scientific). Moreover, this Master Mix has successfully been used with an apple genotyping kit that utilized 17 SSR markers in one reaction, and it was shown to successfully amplify 65 alleles at the same time [44] (a fully heterozygous 8-plex is expected to produce 48 alleles).

Unsurprisingly, not all SSR markers tested showed satisfactory results without PCR optimization (Supplementary Table S2). Also, most of the ECPGR-recommended markers did not work properly under the used PCR conditions (Table 2), and so only two markers (PacA33 and CPSCT026) out of nine were finally chosen. The final selected set of SSR markers was multiplexed into an 8-plex kit that was sufficient to distinguish all 242 unique genotypes tested, which also included the main plum varieties grown in Europe and North America. The adequate diversity of the analyzed sample set was confirmed by the STRUCTURE software version 2.2.4, which identified twenty probable groups (Supplementary Table S5).

The 8-plex kit can be extended by four additional markers amplified simultaneously in the 4-plex. Albeit redundant in our collection (Table 1), 4-plex may be helpful for other collections, the identification of clonal variants, or for the analysis of plum-similar samples with lower ploidy. The kit was therefore also tested in diploid plum-similar species (*P. cerasifera* Ehrh., *P. salicina* Lindl., *P. americana* Marsh.) and in interspecies hybrids that may be phenotypically indistinguishable from hexaploid plums. However, only a limited number of diploid samples (n = 46) was available, not allowing us to make sound conclusions [*Prunus cerasifera* Ehrh. (n = 15), *Prunus salicina* Lindl. (n = 11), and their hybrids or hybrids with *Prunus armeniaca* L. (n = 17), others (n = 3)]. Nonetheless, all samples showed a unique genotype, and at least the information about ploidy may be useful for further evaluations.

Analyses using the 8-plex kit resulted in a median of 36 different alleles per hexaploid sample in our collection. As statistical evaluations of the SSR marker quality in polyploids are difficult [6] and based only on an approximation of the real allele frequency in the population, an alternative approach was followed using the actually observed allele combinations for each marker in the set of 242 samples. The discrimination potential was calculated, indicating how many unique genetic profiles can be, in theory, obtained by combining the identified allele combinations. The 8-plex kit and all 12 markers have expected discrimination potentials of 5.06 × 10^17^ and 4.78 × 10^26^ unique genetic profiles, respectively, implying there is enough discrimination capacity for the thousands of varieties grown worldwide.

In addition to the discrimination potential of the set of SSR markers, the lab-to-lab transferability of the results is no less important. SSR markers are usually reported as being experimentally reproducible and reliably used across different laboratories (for review, see [49]). To standardize fingerprinting by SSR markers, allelic ladders or commonly grown cultivars are used as references to harmonize the binning process, in which relative allele lengths are converted into allele names. However, even assuming good laboratory practices, lab-to-lab comparisons may be sometimes difficult. To help potential users with the implementation of our method, a picture atlas of the typical stutter of individual alleles and their combinations with alleles of similar length is provided (Supplementary Table S3).

Varieties of plums as well as other fruit crops have the strong advantage of being propagated vegetatively. This means that the DNA as well as phenotypic manifestation of the variety is “preserved” from its origin that may have occurred several centuries ago. However, mutations are frequent events and accumulate over time. This may complicate variety identification during genotyping, because clonal variants of the original material may exist since SSR loci may also be affected. This was demonstrated, for example, for the diploid pear variety ‘Rocha’ [50]. It has been proposed that ‘Rocha’ was bred in the 19th century and was greatly propagated and cultivated in Portugal, giving origin to many different phenotypic clonal variants. During the analysis of 79 individuals, they identified 29 different genotypes of ‘Rocha’ differing from the prevailing genotype by 1–6 alleles in 11 SSR markers.

During our hexaploid plum analysis, several samples differing repeatedly by only a few alleles were observed. Some were samples of the same variety but obtained from different sources/trees, while some belonged to different, usually traditional varieties with unknown/uncertain origins. However, it is impossible to set a precise number of alleles that must be different between two samples to distinguish them as being two clonal variants of the same variety or two distinct varieties. In this case, a hierarchical clustering analysis of a tested population and careful analysis of the resulting dendrogram may be helpful to find closely related genotypes in order to delimit likely boundaries between varieties and clonal variants (Figure 4, Supplementary Table S5). In our case, the identified closely related variants (parents vs. offspring) differed at least by 13 alleles, while clonal variants possessed maximally two diverse alleles. Nonetheless, these different allele cut-offs are only approximative, and analyses considering other genotypes may shift these borders and narrow the window between them.

## 5. Conclusions

We developed a kit for the routine genotyping of hexaploid European plums (*P. domestica* L.). The kit used eight verified SSR markers multiplexed in a one-reaction format, which simplifies laboratory workflow, minimizes errors, and saves labor, time, and money, making it ideal for all steps regarding plums, from the characterization of genetic resources [51] to breeding, growing, or controlling. The kit was also successfully tested for other diploid “plums” of various origins and interspecies hybrids; however, further testing is necessary to validate the kit for these varieties.

## Figures and Tables

**Table 1:** A set of 12 SSR markers selected for use in *P. domestica* L. genotyping.

Original SSR Marker	Source Organism	Comment	Reference
AMPA100	*Prunus armeniaca* L.	8-plex	[26]
CPPCT033	*Prunus persica* (L.) Batsch	8-plex	[20]
CPSCT005	*Prunus salicina* Lindl.	8-plex	[22]
CPSCT039	*Prunus salicina* Lindl.	8-plex	[22]
CPSCT042	*Prunus salicina* Lindl.	8-plex	[22]
EMPAS02	*Prunus avium* L.	8-plex	[34]
PacA33 *	*Prunus armeniaca* L.	8-plex	[25]
UDP98-412	*Prunus persica* (L.) Batsch	8-plex	[33]
CPSCT026 *	*Prunus salicina* Lindl.	4-plex	[22]
EMPAS14	*Prunus avium* L.	4-plex	[34]
UCD-CH17	*Prunus avium* L.	4-plex	[32]
UDAp446	*Prunus armeniaca* L.	4-plex	[29]

*: ECPGR-recommended marker.

**Table 2:** Results for ECPGR markers recommended for *P. domestica* L. genotyping.

SSR Marker	Comment
BPPCT007	more than 6 alleles observed
BPPCT014	higher stuttering of longer alleles, alleles differing by 1 nt, binning still possible
BPPCT034	difficult scoring, more than 6 alleles observed, higher stuttering
BPPCT039	weak PCR amplification, +3 cycles needed to obtain a sufficient signal, difficult scoring, more than 6 alleles observed, higher stuttering
BPPCT040	alleles with very low signal observed
CPSCT026	selected, 4-plex
PacA33	selected, 8-plex
UDP96-005	moderate difficulty of scoring, moderate heterogeneity, problematic binning, alleles with very low signal observed
UDP98-407	low heterogeneity

**Table 3:** Selected SSR markers, their combinations in multiplex, and primer characteristics. Primer modifications are in bold.

Primer	Sequence	Final Concentration (µM)	Labeling
**8-plex**			
Blue detection channel: CPPCT033_a + CPSCT042			
CPPCT033_a-F	**GTGAATTCAGCAAACTAGAAACAAAC**	0.350	6-FAM
CPPCT033_a-R	**GCTTTGAAGTGGGTTTGATAATAG**	0.350	-
CPSCT042-F	TGGCTCAAAAGCTCGTAGTG	0.200	6-FAM
CPSCT042-R	CCAACCTTTCGTTTCGTCTC	0.200	-
Green detection channel: UDP98-412 + CPSCT005			
UDP98-412-F1	AGGG**G**AAGTTTCTGCTGCAC	0.175	-
UDP98-412-F2	AG**A**G**G**AAG**C**T**G**CTGCTGCAC	0.175	-
UDP98-412-R	GCTGAAGACGACGATGATGA	0.175	VIC
CPSCT005-F	CTGCAAGCACTG**T**GGATCTC	0.180	VIC
CPSCT005-R	CCCATATTCCCAACCCATTA	0.180	-
Yellow detection channel: EMPaS02 + AMPA100			
EMPaS02-F	CTACTTCCATGATTGCCTCAC	0.180	NED
EMPaS02-R	AACATCCAGAACATCAACACAC	0.180	-
AMPA100-F	TGTTTAGTTGAGGGTAACTTTGG	0.350	NED
AMPA100-R	CCCTTCCTTTTCTGTGTCTCAC	0.350	-
Red detection channel: CPSCT039 + PacA33			
CPSCT039-F	GCCGCA**R**CTCGTAAGGAATA	0.200	PET
CPSCT039-R	TCCAC**Y**GTTGATTACCCTTC	0.200	-
PacA33-F	TCAGTCTCATCCTGCATAC**A**	0.250	PET
PacA33-R	CATGTGGCTCAAGGATCAAA	0.250	-
**4-plex**			
UCD-CH17; EMPAS14; UDAp446; CPSCT026			
UCD-CH17-F	TGGACTTCACTCATTTCAGAGA	0.350	-
UCD-CH17-R	ACTG**Y**AGAGAATTTCCACAACCA	0.350	FAM
EMPAS14-F	TCCGCCATATCACAATCAAC	0.130	VIC
EMPAS14-R	TTCCACACAAAAACCAATCC	0.130	-
UDAp446-F	CCTCCCCCTAGATTTTCAGC	0.120	NED
UDAp446-R	CGTGCTTGGGACATAGATCA	0.120	-
CPSCT026-F	TCTCACACGCTTTCGTCAAC	0.160	PET
CPSCT026-R	AAAAAGCCAAAAGGGGTTGT	0.160	-

**Table 4:** Parameters of selected markers based on the analysis of 242 unique accessions of *P. domestica* L.

Marker	Number of Alleles	Number of Rare Alleles *	Average Number of Alleles Per Genotype	Allele Combinations in 242 Samples	Unique Allele Combinations in % of Samples	Allele Range (nt)	Window Between Markers (nt)
**8-plex**							
Blue detection channel (6-FAM)							
CPPCT033_a	16	8	4.5	143	39.3%	95–129	27
CPSCT042	20	13	4.0	179	55.8%	157–183	
Green detection channel (VIC)							
UDP98-412	26	19	4.4	158	47.9%	87–140	16
CPSCT005	20	11	4.4	183	62.0%	157–209	
Yellow detection channel (NED)							
EMPAS02	15	7	4.4	127	31.4%	107–151	44
AMPA100	16	8	4.7	157	51.2%	196–242	
Red detection channel (PET)							
CPSCT039	27	20	4.3	158	51.7%	89–145	20
PacA33	40	35	4.9	217	81.4%	166–254	
Total	180	121 (67%)	Combined allele	5.06 × 10^17^			
Average	22.5	15	combinations				
**4-plex**							
UCD-CH17	25	15	4.7	182	60.7%	122–169	
EMPAS14	18	11	4.1	132	36.4%	102–199	
UDAp446	44	38	5.2	209	76.4%	120–214	
CPSCT026	28	21	4.8	188	63.6%	165–213	
Total	115	85 (74%)	Combined allele	9.44 × 10^8^			
Average	28.8	21.3	combinations				
**Combined 8-plex + 4-plex**							
Total	295	206 (70%)	Combined allele	4.78 × 10^26^			
Average	24.6	17.2	combinations				

*: allele occurrence below 5%.

**Table 5:** Combinations of markers needed for the discrimination of 242 unique accessions of *P. domestica* L. The UDAp446 marker from the 4-plex is in bold.

Marker	Genotypes Resolved (n; %)		Cumulative	Remaining Genotypes (n)
8-plex markers				
PacA33	197	81.4%		45
+CPSCT039	21	8.7%	90.1%	24
+CPSCT005	8	3.3%	93.4%	16
+AMPA100	6	2.5%	95.9%	10
+CPSCT042	2	0.8%	96.7%	8
All 8 markers	8	3.3%	100.0%	0
	242	100%		
Combined 8-plex + 4-plex markers				
PacA33	197	81.4%		45
+CPSCT039	21	8.7%	90.1%	24
+CPSCT005	8	3.3%	93.4%	16
+AMPA100	6	2.5%	95.9%	10
+UDAp446	3	1.2%	97.1%	7
+CPSCT042	1	0.4%	97.5%	6
All 12 markers	6	2.5%	100.0%	0
	242	100%		

## Data Availability

Data are contained within the article or Supplementary Materials.

## Supplementary Materials

The following supporting information can be downloaded at: https://www.mdpi.com/article/10.3390/plants14152281/s1, Table S1: Plum accessions used in the study; Table S2: Testing results; Table S3: Picture allele atlas; Table S4: Allele occurrence and distribution in markers; Table S5: Structure analysis of a plum germplasm collection.

## References

[1] Das B., Ahmed N., Singh P. (2011). *Prunus* diversity-early and present development: A review. Int. J. Biodivers. Conserv..

[2] Morimoto T., Kitamura Y., Numaguchi K., Akagi T., Tao R. (2019). Characterization of post-mating interspecific cross-compatibility in *Prunus* (Rosaceae). Sci. Hortic..

[3] Okie W.R. (2005). Spring satin plumcot. J. Am. Pomol. Soc..

[4] Neumüller M., Dittrich F., Hartmann W., Hadersdorfer J., Treutter D. (2017). First report on the generation of *Prunus domestica* × *P. armeniaca* interspecific hybrids with hypersensitivity resistance to the Plum pox virus. Acta Hortic..

[5] Zhebentyayeva T., Shankar V., Scorza R., Callahan A., Ravelonandro M., Castro S., DeJong T., Saski C.A., Dardick C. (2019). Genetic characterization of worldwide *Prunus domestica* (plum) germplasm using sequence-based genotyping. Hortic. Res..

[6] Testolin R., Messina R., Cipriani G., De Mori G. (2023). SSR-based DNA fingerprinting of fruit crops. Crop Sci..

[7] Schlötterer C., Tautz D. (1992). Slippage synthesis of simple sequence DNA. Nucleic Acids Res..

[8] Abdallah D., Baraket G., Perez V., Ben Mustapha S., Salhi-Hannachi A., Hormaza J.I. (2019). Analysis of self-incompatibility and genetic diversity in diploid and hexaploid plum genotypes. Front. Plant Sci..

[9] Gasi F., Sehic J., Grahic J., Hjeltnis S.H., Ordidge M., Benedikova D., Blouin-Delmas M., Drogoudi P., Giovannini D., Hofer M. (2020). Genetic assessment of the pomological classification of plum *Prunus domestica* L. accessions sampled across Europe. Genet. Resour. Crop Evol..

[10] Makovics-Zsohár N., Tóth M., Surányi D., Kovács S., Hegedűs A., Halász J. (2017). Simple sequence repeat markers reveal Hungarian plum (*Prunus domestica* L.) germplasm as a valuable gene resource. Hortic. Sci..

[11] Manco R., Basile B., Capuozzo C., Scognamiglio P., Forlani M., Rao R., Corrado G. (2019). Molecular and phenotypic diversity of traditional European plum (*Prunus domestica* L.) germplasm of southern Italy. Sustainability.

[12] Pop R., Monica H., Katalin S., Zănescu M., Sisea C., Catana C., Pamfil D. (2018). Genetic diversity and population structure repeat (SSR) markers. Not. Bot. Horti Agrobot. Cluj-Napoca.

[13] Sehic J., Nybom H., Hjeltnes S.H., Gaši F. (2015). Genetic diversity and structure of Nordic plum germplasm preserved ex situ and on-farm. Sci. Hortic..

[14] Urrestarazu J., Errea P., Miranda C., Santesteban L.G., Pina A. (2018). Genetic diversity of Spanish *Prunus domestica* L. germplasm reveals a complex genetic structure underlying. PLoS ONE.

[15] Xuan H., Ding Y., Spann D., Möller O., Büchele M., Neumüller M. (2011). Microsatellite markers (SRR) as a tool to assist in identification of European plum (*Prunus domestica*). Acta Hortic..

[16] Nybom H., Giovannini D., Ordidge M., Hjeltnes S.H., Grahić J., Gaši F. (2020). ECPGR recommended SSR loci for analyses of European plum (*Prunus domestica*) collections. Genet. Resour..

[17] Antanyniene R., Šikšnianiene J.B., Stanys V., Frercks B. (2023). Fingerprinting of plum (*Prunus domestica*) genotypes in Lithuania using SSR Markers. Plants.

[18] Meland M., Frøynes O., Fotirić Akšić M., Pojskić N., Kalamujić Stroil B., Miralem M., Konjić A., Gasi F. (2024). Genetic characterization of European plum (*Prunus domestica* L.) accessions from Norway using ECPGR-selected SSR markers. Agronomy.

[19] Cipriani G., Lot G., Huang W.G., Marrazzo M.T., Peterlunger E., Testolin R. (1999). AC/GT and AG/CT microsatellite repeats in peach [*Prunus persica* (L) Batsch]: Isolation, characterisation and cross-species amplification in Prunus. Theor. Appl. Genet..

[20] Aranzana M.J., Garcia-Mas J., Carbo J., Arús P. (2002). Development and variability analysis of microsatellite markers in peach. Plant Breed..

[21] Dirlewanger E., Cosson P., Tavaud M., Aranzana M., Poizat C., Zanetto A., Arús P., Laigret F. (2002). Development of microsatellite markers in peach [*Prunus persica* (L.) Batsch] and their use in genetic diversity analysis in peach and sweet cherry (*Prunus avium* L.). Theor. Appl. Genet..

[22] Mnejja M., Garcia-Mas J., Howad W., Badenes M.L., Arús P. (2004). Simple-sequence repeat (SSR) markers of Japanese plum (*Prunus salicina* Lindl) are highly polymorphic and transferable to peach and almond. Mol. Ecol. Notes.

[23] Clarke J., Tobutt K. (2003). Development and characterization of polymorphic microsatellites from *Prunus avium* ‘Napoleon’. Mol. Ecol. Notes.

[24] Mnejja M., Garcia-Mas J., Howad W., Arús P. (2005). Development and transportability across *Prunus* species of 42 polymorphic almond microsatellites. Mol. Ecol. Notes.

[25] Decroocq V., Favé M., Hagen L.S., Bordenave L., Decroocq S. (2003). Development and transferability of apricot and grape EST microsatellite markers across taxa. Theor. Appl. Genet..

[26] Hagen L., Chaib J., Fady B., Decroocq V., Bouchet J.P., Lambert P., Audergon J.M. (2004). Genomic and cDNA microsatellites from apricot (*Prunus armeniaca* L.). Mol. Ecol. Notes.

[27] Lalli D.A., Abbott A.G., Zhebentyayeva T.N., Badenes M.L., Damsteegt V.D., Polak J.W., Krška B., Salava J. (2008). A genetic linkage map for an apricot (*Prunus armeniaca* L.) BC1 population mapping plum pox virus resistance. Tree Genet. Genomes.

[28] Lopes M.S., Sefc K.M., Laimer M., Machado A.D. (2001). Identification of microsatellite loci in apricot. Mol. Ecol. Notes.

[29] Messina R., Lain O., Marrazzo M.T., Cipriani G., Testolin R. (2004). New set of microsatellite loci isolated in apricot. Mol. Ecol. Notes.

[30] Soriano J.M., Domingo M.L., Zuriaga E., Romero C., Zhebentyayeva T., Abbott A.G., Badenes M.L. (2012). Identification of simple sequence repeat markers tightly linked to plum pox virus resistance in apricot. Mol. Breed..

[31] Sosinski B.R., Gannavarapu M., Hager L.D., Beck L., King G.J., Ryder C.D., Rajapakse S., Baird W.V., Ballard R., Abbott A.G. (2000). Characterization of microsatellite markers in peach [*Prunus persica* (L.) Batsch]. Theor. Appl. Genet..

[32] Struss D., Ahmad R., Southwick S.M., Boritzki M. (2003). Analysis of Sweet Cherry (*Prunus avium* L.) Cultivars Using SSR and AFLP Markers. J. Am. Soc. Hortic. Sci..

[33] Testolin R., Marrazzo T., Cipriani G., Quarta R., Verde I., Dettori M.T., Pancaldi M., Sansavini S. (2000). Microsatellite DNA in peach (*Prunus persica* L. Batsch) and its use in fingerprinting and testing the genetic origin of cultivars. Genome.

[34] Vaughan S., Russell K. (2004). Characterization of novel microsatellites and development of multiplex PCR for large-scale population studies in wild cherry, *Prunus avium*. Mol. Ecol. Notes.

[35] Vendramin E., Dettori M.T., Giovinazzi J., Micali S., Quarta R., Verde I. (2007). A set of EST-SSRs isolated from peach fruit transcriptome and their transportability across *Prunus* species. Mol. Ecol. Notes.

[36] Callahan A.M., Zhebentyayeva T.N., Humann J.L., Saski C.A., Galimba K.D., Georgi L.L., Scorza R., Main D., Dardick C.D. (2021). Defining the ‘HoneySweet’ insertion event utilizing NextGen sequencing and a de novo genome assembly of plum (*Prunus domestica*). Hortic. Res..

[37] Pritchard J.K., Stephens M., Donnelly P. (2000). Inference of population structure using multilocus genotype data. Genetics.

[38] Earl D.A., vonHoldt B.M. (2012). STRUCTURE HARVESTER: A website and program for visualizing STRUCTURE output and implementing the Evanno method. Conserv. Genet. Resour..

[39] Evanno G., Regnaut S., Goudet J. (2005). Detecting the number of clusters of individuals using the software STRUCTURE: A simulation study. Mol. Ecol..

[40] Huang K., Dunn D.W., Ritland K., Li B.G. (2020). POLYGENE: Population genetics analyses for autopolyploids based on allelic phenotypes. Methods Ecol. Evol..

[41] Sokal R.R., Michener C.D. (1958). A statistical method for evaluating systematic relationships. Univ. Kans. Sci. Bul..

[42] Liu Y., Zhao Q., Li T., Teng C., Peng H., Yao Z., Fang Z., Zhou J., Yang X., Qiao J. (2024). Availability Evaluation and Application of MNP (Multiple Nucleotide Polymorphism) Markers in Variety Identification of Chrysanthemum. Horticulturae.

[43] Phillips A.R. (2024). Variant calling in polyploids for population and quantitative genetics. Appl. Plant Sci..

[44] Cmejlova J., Rejlova M., Paprstein F., Cmejla R. (2021). A new one-tube reaction kit for the SSR genotyping of apple (*Malus* × *domestica* Borkh.). Plant Sci..

[45] Zurn J.D., Nyberg A., Montanari S., Postman J., Neale D., Bassil N. (2020). A new SSR fingerprinting set and its comparison to existing SSR- and SNP-based genotyping platforms to manage *Pyrus* germplasm resources. Tree Genet. Genomes.

[46] Akin M., Nyberg A., Postman J., Mehlenbacher S., Bassil N. (2017). A multiplexed microsatellite fingerprinting set for hazelnut cultivar identification. Eur. J. Hortic. Sci..

[47] Chambers A., Carle S., Njuguna W., Chamala S., Bassil N., Whitaker V., Barbazuk W., Folta K. (2013). A genome-enabled, high-throughput, and multiplexed fingerprinting platform for strawberry (*Fragaria* L.). Mol. Breed..

[48] Bassil N., Bidani A., Nyberg A., Hummer K., Rowland L. (2020). Microsatellite markers confirm identity of blueberry (*Vaccinium* spp.) plants in the USDA-ARS National Clonal Germplasm Repository collection. Genet. Resour. Crop Evol..

[49] Vieira M.L., Santini L., Diniz A.L., Munhoz C.D. (2016). Microsatellite markers: What they mean and why they are so useful. Genet. Mol. Biol..

[50] Queiroz Á., Bagoin Guimarães J., Sánchez C., Simões F., Maia de Sousa R., Viegas W., Veloso M.M. (2019). Genetic Diversity and Structure of the Portuguese Pear (*Pyrus communis* L.) Germplasm. Sustainability.

[51] Anglin N.L., Wenzl P., Azevedo V., Lusty C., Ellis D., Gao D. (2025). Genotyping Genebank Collections: Strategic Approaches and Considerations for Optimal Collection Management. Plants.

